# Role of tourniquet release timing on blood loss and functional outcomes in total knee arthroplasty: Insights from a low‐ and middle‐income country

**DOI:** 10.1002/jeo2.12075

**Published:** 2024-07-24

**Authors:** Muhammad Ahmed Ghazni Khan, Mohammad Ahsan Sulaiman, Marij Zahid, Suresh Kumar, Tashfeen Ahmad

**Affiliations:** ^1^ Department of Surgery Aga Khan University Hospital Karachi Pakistan

**Keywords:** blood loss, functional outcome, total knee arthroplasty, tourniquet

## Abstract

**Purpose:**

To identify the effect of releasing a tourniquet before versus after wound closure in total knee arthroplasty (TKA) on blood loss, functional outcome and postoperative complications.

**Methodology:**

A prospective cohort study was conducted including 53 patients from May 2023 to September 2023. All patients underwent unilateral TKA and were divided into two groups based on surgeon preference of deflating tourniquet, Group A consisted of patients in whom the tourniquet was deflated before wound closure for haemostasis and Group B consisted of patients in which tourniquet was deflated after wound closure and compressive dressing. Blood loss was evaluated via intraoperative blood loss (the number of soaked sponges/gauzes, blood in a suction bottle, total output in a suction bottle—irrigation used) and on‐field blood loss and calculated blood loss (Using Gross and Meunier's formula). The Functional outcome was evaluated using Knee injury and osteoarthritis score‐42 questions. Early postoperative complications and differences in the requirement of blood transfusions were also assessed.

**Results:**

There was a significant difference in intraoperative blood loss between the two groups. The median intraoperative blood loss was 135 mL (interquartile range [IQR]: 90–149) in Group A and 56.2 mL (IQR: 45–68) in Group B (*p* value: 0.001). However, no difference was found between the groups in calculated blood loss using Gross and Meunier's formula. The median calculated blood loss was 439 mL (IQR: 450–813) in Group A and 508 mL (IQR: 226–671) in group B (*p* value: 0.981). There was no significant difference between the groups in blood transfusion requirements or functional outcomes.

**Conclusion:**

Based on our results, we conclude that the intraoperative blood loss in TKA is significantly different between the groups but only represents a fraction of true blood loss (23%). The timing of releasing the tourniquet does not affect functional outcomes, blood transfusion and postoperative morbidity; hence, any time can be opted as per surgeon preference.

**Level of Evidence:**

Level II, prospective comparative study.

**Abbreviations**
HB: haemoglobin
Hct: haematocrit
KOOS‐42: Knee injury and osteoarthritis score‐42 questions
OA: osteoarthritis
TKA: total knee arthroplasty

## INTRODUCTION

Osteoarthritis (OA) of the knee is one of the most common conditions, and its pooled global prevalence is estimated to be as high as 22.9% in individuals over 40 years of age [5]. In the advanced stage, it can severely affect a patient's quality of life, leading to multiple health problems due to persistent pain, limited mobility and chronic use of nonsteroidal anti‐inflammatory drugs [12, 16].

One of the most accepted treatment options for advanced knee OA is total knee arthroplasty (TKA) [19], because of its safety and beneficial effects on a patient's quality of life, health status and mobility including decreasing the chronic use of analgesics and related side effects [13, 24]. The number of total knee arthroplasties performed is increasing exponentially each day [6], which led us to focus on the postoperative complications following TKA. Perioperative blood loss and iatrogenic injury to major blood vessels are some of the major complications in TKA [10].

It is believed that the use of a tourniquet significantly reduces operative blood loss, enhances visualisation, decreases surgical time and eases the cementing of the prosthesis [1, 23]. However, tourniquet application has also been shown to increase complications like postoperative thigh pain, wound complications, neuromuscular injuries and increased thrombotic events [8, 17]. To decrease the tourniquet time and tourniquet‐related complications, some surgeons prefer to release the tourniquet after cementation before wound closure for better assessment of haemostasis and to prevent unseen complications, while others prefer to open the tourniquet after wound closure. However, there is still controversy in international literature as to which practice is superior to the other. Some studies have shown that deflating the tourniquet before wound closure increases blood loss but decreases complication rates, while others have shown no significant difference [2, 9, 22, 25]. Whether to remove the tourniquet before or after wound closure in TKA is still under debate and mostly depends on surgeon preference. Conducting this study in Pakistan, a low‐ to middle‐income country, fills a critical gap in the existing literature by addressing regional disparities. While studies from developed countries have contributed valuable evidence on tourniquet release timing in TKA, their findings may not fully translate to low‐ or middle‐income countries like Pakistan due to differences in healthcare infrastructure, socioeconomic factors and patient population differences.

### Primary objective

The objective of this study was to identify the effect of releasing the tourniquet before wound closure versus after wound closure in TKA on blood loss, measured by intraoperative blood loss (mL), calculated blood loss and hidden blood loss.

### Secondary objective

The study also aimed to find differences in postoperative functional outcomes and complications between the two groups.

## MATERIALS AND METHODS

It was a single‐centred, prospective cohort study including patients who underwent unilateral total knee replacement at a tertiary care centre from May 2023 to September 2023. This study was conducted after approval from the institutional ethical review committee (ERC 2023‐8344).

### Inclusion criteria

All patients above 30 years of age and diagnosed with end‐stage knee OA using clinical and radiological parameters and underwent unilateral TKA using a tourniquet were included after formal informed consent.

### Exclusion criteria

Patients who did not consent to enrol or who underwent bilateral, revision or unicompartmental knee arthroplasty were excluded from the study. Additionally, patients with known coagulation disorders or those who were lost to follow‐up were also excluded

Demographic variables such as age, gender, body mass index (BMI) and comorbid conditions were recorded on a prestructured proforma. Intraoperative parameters such as intraoperative blood loss, operative time and time to release of tourniquet were also recorded. The cohort was divided into two groups based on surgeon preference regarding when to open the tourniquet; the exposed (Group A) consisted of patients in whom the tourniquet was released before the closure of the wound and after the cementation of the implant, while the unexposed (Group B) included patients in whom tourniquet was released after the closure of the wound.

### Surgical procedure

Intraoperatively a tourniquet was applied around the proximal thigh before sterile preparation and draping of the patient. The anaesthesia team preoperatively decided the use of general or spinal anaesthesia. A dose of intravenous tranexamic acid 15 mg/kg and antibiotics were given preoperatively just after induction. In all cases, the tourniquet was inflated to 100 mm Hg above the systolic pressure before the skin incision. All procedures were done using the medial parapatellar approach by one of the five arthroplasty‐trained surgeons with more than 5 years of experience. The prosthesis utilised was posterior stabilised and cemented in all cases. No postoperative drain was placed in any of the patients. Timing of tourniquet deflation was based on surgeon preference, and patients were divided into two groups based on the timing as detailed above.

Perioperative parameters including intraoperative blood loss, tourniquet deflation timings and blood transfusion requirement were recorded for each patient. Postoperative haemoglobin and haematocrit were repeated on the first postoperative day in each case and recorded. Functional outcome was assessed using Knee injury and osteoarthritis score‐42 questions (KOOS‐42) Score both preoperatively and at 3 months postsurgery follow‐up. Patients were also assessed for any possible complications at 3‐month follow‐up.

### Outcome measures

Intraoperative blood loss was calculated by the number of soaked sponges/gauzes, blood in the suction bottle (total output in suction bottle‐irrigation used) and on‐field blood loss.

Calculated blood loss was recorded using two different methods:

Method 1: Estimated by gross method, Gross [7]. This formula calculates total blood loss via a difference in haematocrit level:

V loss total: BV × (Hct preoperative − Hct f)/Hct average,

V loss: Blood volume loss (mL),

Hct f: Minimum allowable haematocrit,

Hct Av: Average of initial and minimum haematocrit,

BV: The patient's blood volume before surgery, blood volume was calculated based on the formula given by Nadler et al. [15],

BV: K1 × H3 +K2 × *W* + K3.

**Table:** 

Constant	Male	Female
K1	0.3669	0.3561
K2	0.03219	0.03308
K3	0.6041	0.1833

Method 2: Haemoglobin drop, Meunier's formula [14].

Blood loss by haemoglobin dilution method: BV × (haemoglobin preoperative − Haemoglobin postoperative)/haemoglobin postoperative.

Hidden blood loss: Calculated blood loss minus Intraoperative blood loss.

### Statistical analysis

Stata version 15 was used for data entry and analysis. Quantitative variables (age, height, weight and BMI) were expressed as mean ± standard deviation/median (IQR) and qualitative variables (gender, comorbid conditions) were expressed as frequencies. Comparison of continuous variables KOOS and blood loss both pre‐ and post‐TKA was done using the Wilcoxin sign rank test, as the majority of the predictor variables were skewed. Independent Sample *t* test was used to compare the KOOS score between Group A and B. Pearson co‐relation coefficient was used to analyse any association between continuous variables (e.g. age) with the outcome (KOOS, blood loss). A *p* value of <0.05 was considered significant.

## RESULTS

A total of 53 patients were included in the study. Ten patients were lost to follow‐up and consequently excluded. Out of 43 patients, 20 were in Group A (tourniquet released before wound closure) and 23 were in Group B (tourniquet released after wound closure). The mean age of all the patients was 60.5 ± 9 years. The patients in both groups were comparable regarding age, gender distribution and preoperative haemoglobin. The Mean BMI was 29.31 ± 5.6 in Group A and 32.6 ± 4.8 in Group B, showing a statistically significant difference in BMI between the two groups (*p* = 0.04) (see Table 1).

**Table 1:** Base line demographic data and scores of subgroups.

	Tourniquet opening		
Variable	Before	After	*P*‐value
Age in years ± SD	61.85 ± 9.4	60.2 ± 9.9	0.587
BMI ± SD	29.31 ± 5.6	32.6 ± 4.8	0.04
Gender: Male (%)	6 (30%)	6 (26.09%)	0.775
Female (%)	14 (70%)	17 (73.9%)	
Torniquet time min (SD)	67 (5.83)	76 (6.1)	0.0001
Preoperative HB (gm/dL)	12.8 ± 1.8	12.5 ± 1.2	0.6
Preoperative Hct (gm/dL)	39.5 ± 4.7	39.4 ± 3.5	0.92
KOOS admission (mean ± SD)	27.24 ± 7.7	26.82 ± 5.8	0.841
KOOS 3 months	71.505 ± 6.3	73.18 ± 7.0	0.371
Laterality: Right (%)	12 (60)	12 (52.17)	0.606
Left (%)	8 (40)	11 (47.8)	

Abbreviations: BMI, body mass index; HB, haemoglobin; Hct, haematocrit; KOOS, Knee injury and osteoarthritis score.

Tourniquet time was 67 ± 5.8 min in Group A and 76 ± 6.1 min in Group B with a statistically significant difference (*p* = 0.001). The median intraoperative blood loss was 135 mL (IQR: 90–149) in Group A and 56.2 mL (IQR: 45–68) in Group B, which was statistically significant (*p* = <0.001).

**Figure 1:**
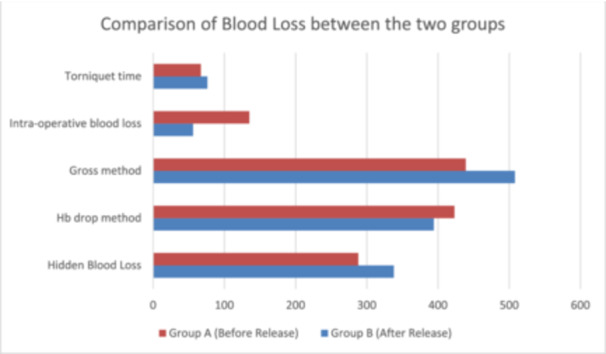
Comparison of blood loss between the two groups. Hb, haemoglobin.

The blood loss was also calculated using two different methods, that is, Gross and Meunier's method. No statistically significant difference was found in the calculated blood loss by these methods (see Table 2). However, there was a significant difference between intraoperative blood loss and calculated blood loss in both groups (*p* = <0.001). The intraoperative blood loss only represented 23% of the calculated blood loss in both groups (see Figure 1).

**Table 2:** Changes in the parameters of the group.

	Tourniquet opening		
Variable	Before	After	*P*‐value
Postoperative HB (gm/dL)	11.14 ± 1.3	11.18 ± 1.4	0.98
Postoperative Hct (gm/dL)	35.3 ± 3.9	35.4 + 3.3	0.941
Intraoperative blood loss (mL) (IQR)	135 (90–149)	56.2 (45–68)	0.001
Transfusion PC (patients)	4 PC (3 pts)	2 PC (2pts)	0.194
Calculated blood loss (mL)			
Gross method (IQR)	439 (450–813)	508 (226–671)	0.981
Hb drop method (IQR)	423 (228–801)	394 (293–589)	0.697
Hidden blood loss (IQR)	288 (138–711)	338 (188–551)	0.237

Abbreviations: IQT, interquartile range; PC, pack cell.

**Figure 2:**
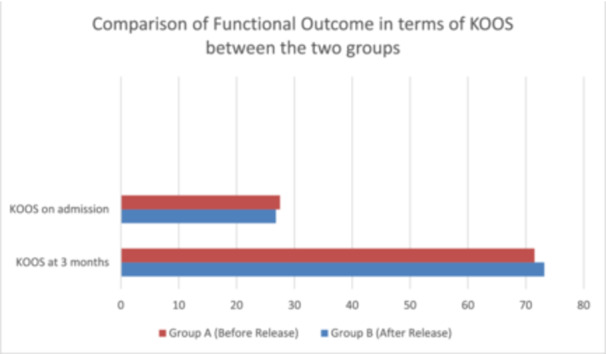
Comparison of functional outcome in terms of Knee injury and osteoarthritis score (KOOS) between the groups.

There was no statistically significant difference in transfusion between the two groups (*p* = 0.19). Functional outcomes measured using KOOS‐42 at 3 months were comparable between both the groups, with no statistically significant difference (*p* = 0.37) (see Figure 2).

To check for any confounding variables like age and BMI on the effect of tourniquet timing and blood loss, group stratification was done and then checked for any confounding, no change in association with the outcome was noted. To measure the effect of tourniquet opening timing and intraoperative blood loss, univariate regression analysis was done and the regression coefficient was 33.03 (*p* < 0.001), indicating that, on average, the intraoperative blood loss was 33 mL more in patients in Group A than patients in Group B keeping other predictor variables constant.

The study also attempted to see any complications such as deep venous thrombosis, tourniquet palsy and wound complications, however, the study was not powered enough to detect uncommon complications.

## DISCUSSION

The study showed tourniquet release before wound closure for haemostasis, significantly increased intraoperative blood loss which is consistent with other studies [11, 22]. This is because of bleeding from the soft tissue and bone as the tourniquet was released before the wound closure; second, Alecelik et al. reported more bleeding after deflation of the tourniquet because of increased fibrinolytic activity [1]. The study also found decreased intraoperative blood loss in Group B where the tourniquet was deflated after wound closure. This is because closed wounds and firmly applied dressing provide a local compressive effect in controlling the oozing from the tissues.

Different methods were proposed in the literature to calculate blood loss in TKA. In this study, blood loss was calculated using the Gross method and Meunier's formula (haemoglobin drop method) and did not find any statistically significant difference in calculated blood loss between the two groups. Our study findings are similar to literature evidence including a meta‐analysis by Tai et al. [20, 21], however in a meta‐analysis by F Zan et al. and Huang et al., they reported that there is increased calculated blood loss in opening tourniquet before wound closure [11, 22]. One possible explanation for no significant calculated blood loss difference in the groups despite the significant difference in intraoperative blood loss is that haemostasis done in the before‐release group will decrease postoperative blood loss, whereas in the after‐release group, leaving small bleeding source for primary haemostasis and increased blood flow after deflation likely increase postoperative blood loss.

Previous studies showed that calculated blood loss is more accurate than intraoperative blood loss [18]. Result of this study showed that there is a significant difference between intraoperative blood loss and calculated blood loss in both groups which is consistent with other studies, Zhang and Tie [21, 23]. Considering this difference, intraoperative blood loss only represents a fraction of the true blood loss. In this study, intraoperative blood only represents 23% of calculated blood loss. Therefore, the operating surgeon should consider a low threshold for blood transfusion to prevent early postoperative complications. This study also showed that there is substantial hidden blood loss almost 50%–65% in TKA.

Regarding early postoperative patient‐reported functional outcomes, calculated with KOOS‐42 at 3 months after the surgery no difference was found between the two groups, which is similar to other studies done by Beckers et al. [3] and Çi̇nka et al [4]. The 3‐month follow‐up period is selected to capture the early postoperative period when significant improvements in patient reported outcome measures are typically observed. Additionally, a 3‐month interval facilitates timely intervention if any suboptimal outcome arises, enabling healthcare providers to address issues promptly and optimise patient outcomes. In our study, no postoperative complication was noticed in either group.

### Limitations

This study was a single‐centre study with a limited population and only included unilateral primary TKA patients of different surgeons, which may have affected the final functional outcome. Knee OA is more common in the female population [5] hence, the female population is larger compared to the male population in both groups. Moreover, blood loss may continue for several days after surgery; this study only considered Hb/Hct levels 24 h after surgery. In our study, no postoperative complications were noticed in either group likely because this study was not powered enough to detect such uncommon complications of TKA.

## CONCLUSION

This study contributes essential insight into tourniquet release timings in TKA concerning blood loss and early functional outcomes. In consideration of our study, we concluded that intraoperative blood loss was significantly different between the groups but only represented a fraction of true blood loss and should prompt the surgeon to anticipate timely blood transfusions. There is no significant difference in calculated blood loss and functional scores at 3‐month intervals opening tourniquet before versus after wound closure. We recommend that the timing of releasing the tourniquet does not affect functional outcomes, blood transfusion and postoperative morbidity; hence any time can be opted as per surgeon's preference.

## AUTHOR CONTRIBUTIONS

All authors contributed to the study's conception and design. Material preparation, data collection and analysis were performed by Muhammad Ahmed Ghazni Khan, Marij Zahid, Ahsan Sulaiman and Suresh Kumar. The first draft of the manuscript was written by Ahmed Ghazni Khan and all authors commented on previous versions of the manuscript. All authors read and approved the final manuscript. All work was done under the kind supervision of Tashfeen Ahmed.

## CONFLICT OF INTEREST STATEMENT

The authors declare no conflict of interest.

## ETHICS STATEMENT

The study was conducted after ethical committee approval from the department of ERC, Aga Khan University Hospital. ERC Number: (ERC 2023‐8344).

## Data Availability

The data that support the findings of this study are available from the corresponding author upon request.
